# Single-view multi-human pose estimation by attentive cross-dimension matching

**DOI:** 10.3389/fnins.2023.1201088

**Published:** 2023-07-19

**Authors:** Wei Tian, Zhong Gao, Dayi Tan

**Affiliations:** Institute of Intelligent Vehicles, School of Automotive Studies, Tongji University, Shanghai, China

**Keywords:** attentive learning, multi-person pose estimation, single-image pose estimation, keypoint prediction, cross-dimension matching

## Abstract

Vision-based human pose estimation has been widely applied in tasks such as augmented reality, action recognition and human-machine interaction. Current approaches favor the keypoint detection-based paradigm, as it eases the learning by circumventing the highly non-linear problem of direct regressing keypoint coordinates. However, in such a paradigm, each keypoint is predicted based on its small surrounding region in a Gaussian-like heatmap, resulting in a huge waste of information from the rest regions and even limiting the model optimization. In this paper, we design a new k-block multi-person pose estimation architecture with a voting mechanism on the entire heatmap to simultaneously infer the key points and their uncertainties. To further improve the keypoint estimation, this architecture leverages the SMPL 3D human body model, and iteratively mines the information of human body structure to correct the pose estimation from a single image. By experiments on the 3DPW dataset, it improves the state-of-the-art performance by about 8 mm on MPJPE metric and 5 mm on PA-MPJPE metric. Furthermore, its capability to be employed in real-time provides potential applications for multi-person pose estimation to be conducted in complex scenarios.

## 1. Introduction

Vision-based human pose estimation has been favored in tasks of augmented reality, action recognition, human-machine interaction, etc. However, estimating human poses from a single image is a persistent challenge for the research community. In traditional algorithms, manually designed human body models are adopted to obtain local representations and global pose structures. However, the complexity of the human pose is far beyond the representation ability of hand-crafted features. In recent years, various human pose estimation technologies have been progressed driven by deep learning algorithms and large datasets.

The current mainstream 2D Human Pose Estimation (HPE) models can be divided into two categories: regression-based method and detection-based method. The former attempts to learn the direct mapping from an image to human keypoint (e.g, joint) coordinates (Toshev and Szegedy, 2014), which is yet a highly nonlinear problem and difficult to learn. The latter has dominated HPE for years due to high performance and intends to predict location heatmaps of parts or key points (Newell et al., 2016; Chu et al., 2017). However, the heatmaps are typically with low feature resolution and each keypoint only focuses on a small local region, resulting in a large waste of propagated gradients from the rest regions during model optimization.

Considering that current methods do not make full use of the information of human body structure, we propose a new k-block human pose estimation approach. Given a forecasted heatmap, this approach employs a voting mechanism over the entire heatmap to calculate keypoint coordinates and their corresponding uncertainties. Thus, compared to the traditional form, more feature information can be utilized through the increased number of back-propagated gradients, and non-informative key points (e.g., by occlusion) will be given less attention during learning.

Due to the lack of depth information, the traditional 2D pose estimation often yields keypoint ambiguity. However, the human body structure based on 3D coordinates can better alleviate this problem. Leveraging the Skinned Multi-Person Linear (SMPL) 3D structure model of human body (Loper et al., 2015), we design an iterative coordinate matching strategy between 2D and 3D key points. The matching is optimized by using the Singular Value Decomposition (SVD) algorithm. The 2D coordinates can be corrected based on the predicted 3D key points and the optimized corresponding Euclidean transforms.

Compared with other human pose estimation schemes, we focus on mining the prior structure information of the human body itself and use the information of key points to reconstruct the pose model. With the new designed k-block module and corresponding optimization algorithm, the human body pose information can be iteratively corrected and the final output is based on the combination of the predicted human 2D/3D pose estimation.

## 2. Related works

### 2.1. 2D human pose estimation

As aforementioned, the direct regression learning of keypoint coordinates is difficult because it is a highly nonlinear problem, which lacks learning robustness. In comparison, the heatmap learning has a dense pixel information supervision, but the resolution of the heatmap is usually low due to downsampling operations such as pooling and strided convolution in the model, which limits the accuracy of the final estimated coordinates. A typical effort to this problem is the design of Hourglass module (Newell et al., 2016). It uses an hourglass-shaped model to gradually restore the features compressed in high-dimensional space to the original scale. Detail information such as faces and hands are captured by local features, which are restored and fused in the corresponding heatmaps with the same dimensions of features. Further efforts such as data stream adjustment (Bulat et al., 2020) and high-resolution (Sun et al., 2019) are also proposed to improve the network efficiency.

In addition to the keypoint detection, another problem that should be faced in the multi-person pose estimation is how to divide a large number of recognized pose key points into corresponding human bodies. The existing solutions are mainly divided into the top-down and the bottom-up paradigms. The former is achieved with a two-stage pipeline, which firstly employs off-the-shelf detectors on the input image to locate region of interests (RoI, denoted by bounding boxes) of human bodies, which are then individually processed by single-person pose estimators. But such approaches may be suboptimal since the pose estimation results are significantly affected by the detection accuracy, the focus of these methods is on the exploration of more efficient detectors (He et al., 2017; Ren et al., 2017). In contrast, the bottom-up methods firstly predict the key points of all persons in the image and then group them into different human bodies. The difficulty lies in how to correctly assemble the joint points. A typical approach is the OpenPose (Cao et al., 2017). It uses the Part Affinity Fields (PAF) module to predict the Part Confidence Maps and Part Affinity Fields on the entire image, which are further matched based on the learned local association fields. In other approaches, Newell et al. performed simultaneous detection and grouping with the Associative Embedding (Newell et al., 2017). They designed a new deep network structure to generate location heatmaps and associative embedding tags for each joint, distinguishing between different human bodies by tags. Although the processing speed of bottom-up methods is relatively fast and even real-time applicable (Cao et al., 2017; Nie et al., 2018), their performance is greatly affected by the complex backgrounds or occlusions. Therefore, motion information has been considered in recent works (Ohashi et al., 2020; Wang et al., 2020), which yet require video frames instead of a single image as inputs.

### 2.2. 3D human pose estimation

In mainstream models, the 3D human pose estimation is defined as the estimation of 3D human joint points. Related methods are mainly divided into two strategies: one-stage estimation and two-stage estimation. The one-stage methods directly estimate 3D poses from the input image in the presentations such as 3D heatmaps (Pavlakos et al., 2017), position maps (Sun Y. et al., 2021), and depth information (Liu et al., 2021). In contrast, the two-stage methods firstly estimate 2D human poses and then uplift them to the 3D space via pre-learned structural information (Zhou et al., 2016, 2017) or regression models (Martinez et al., 2017; Sun et al., 2017). Since two-stage methods are highly dependent on accurate 2D pose estimators, the combination of powerful backbone networks (Simonyan and Zisserman, 2015; Sun S. et al., 2021) became a trend in achieving impressive performance. However, as the human body structure information is implicitly modeled by neural networks, there is no guarantee that the output 3D skeleton in these methods is consistent with the real ones.

Aside from the 3D skeletons, the prior statistics about human body structure have also drawn increased research attention. A representative is the SMPL human body model (Loper et al., 2015), which is utilized to parameterize the output targets in model-based 3D pose estimation methods. Compared with model-free methods, these approaches directly predict controllable parameters, which facilitates an end-to-end 3D pose estimation without secondary adjustment, such as the SMPLify model proposed by Bogo et al. (2016). Since the mapping from an image to the shape space and the relative rotation of body parts is hard to learn, forms of intermediate representations and supervision are chosen to alleviate this problem, such as contours, semantic part segmentation, and 2D heatmaps. For example, Kanazawa et al. (2018) designed the adversarial priors and iterative error feedback (IEF) loops to reduce the difficulty of regression. Arnab et al. (2019) exploited temporal context information. Guler and Kokkinos (2019) used partial voting expressions and post-processing to improve regression networks. Kolotouros et al. (2019) leveraged an optimization paradigm to provide additional 3D supervision from unlabeled images. The hybrid inverse kinematics solution (HybrIK) (Li et al., 2021) leveraged the twist-and-swing decomposition to transform the 3D joints to shape estimation via both Kinematics and inverse Kinematics modeling and circumvented direct learning the abstract parameters of the general human body models.

**Figure 1:**
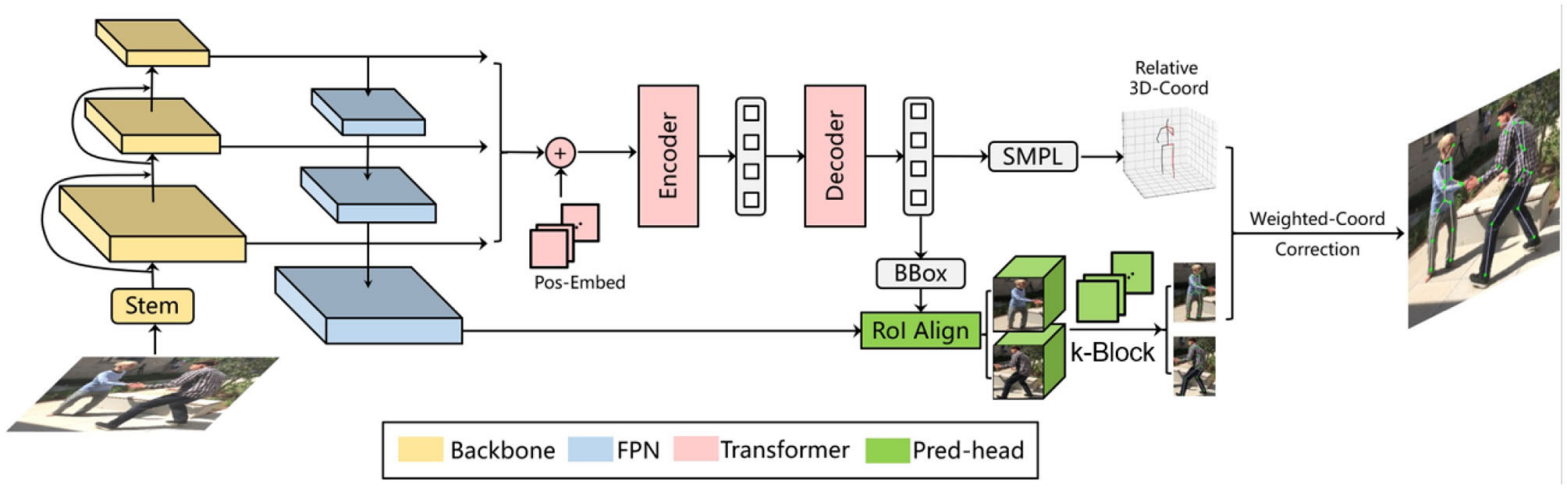
An overview of proposed multi-person pose estimation framework. Reproduced with permission from the official 3DPW benchmark, available at https://virtualhumans.mpi-inf.mpg.de/3DPW/.

In this paper, we propose a novel monocular multi-person pose estimation framework by exploiting the advantages of both 2D and 3D strategies. For backbone, this framework employs the Deformable DETR model (Zhu et al., 2021) (left part of Figure 1). It serves as a multi-person detector as well as a provider of reference regions and image features for the k-block module, which covers the entire heatmap information by a voting mechanism. Additionally, the k-block introduces uncertainties to 2D keypoint estimation, so that occluded joint points are given lower weights in the learning process, as they are less informative and can be inaccurately estimated, resulting in higher uncertainties. We also leverage an SMPL-based parametric model with a 2D–3D iterative optimization process. The core of our optimization algorithm is to estimate the optimal transform matrix and depths through iterative fitting between 2D and 3D relative coordinates. In this way, an accurate pose estimation can be obtained step by step without requring depth information.

## 3. Proposed method

### 3.1. 2D human characterization based on k-block structure

As previously introduced, the existing detection-based 2D pose estimation paradigm is designed to predict the location heatmap of key points, but is limited by the insufficient computational resolution. Moreover, most values on the heatmap are set to zero except for small local region surrounding the joints (**Figure 4B**), thus having no effect on the estimation of joint point coordinates. This fact forces a lot of back-propagated gradients to suppress predictions at non-joint positions, not only leading to a less efficient overall learning, but also making the model preferentially predict zero values.

**Figure 2:**
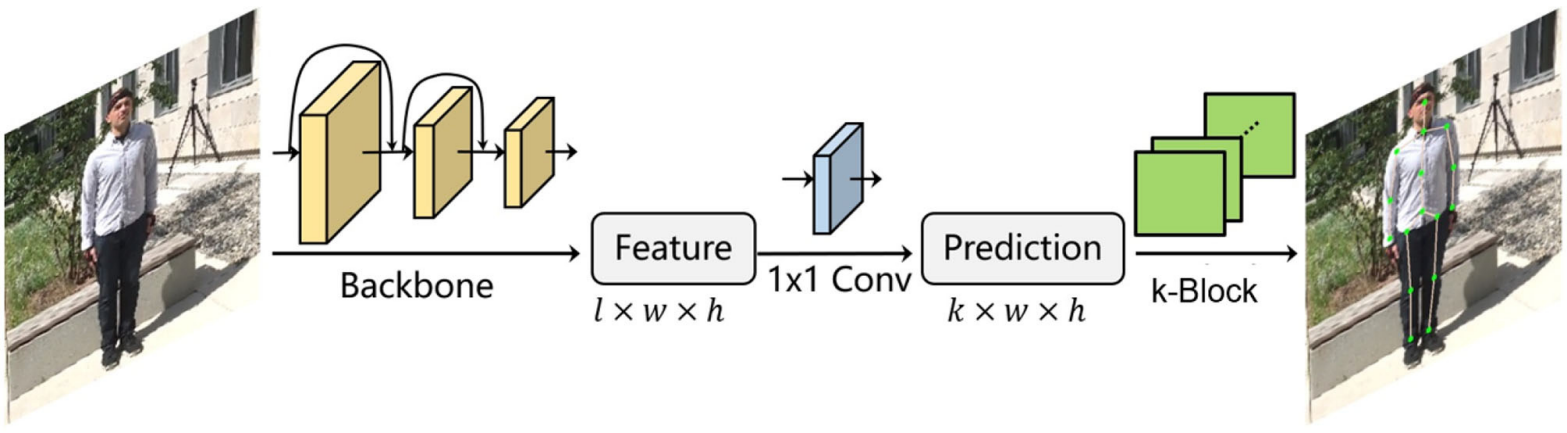
k-block-based 2D single-person pose estimation.

**Figure 3:**
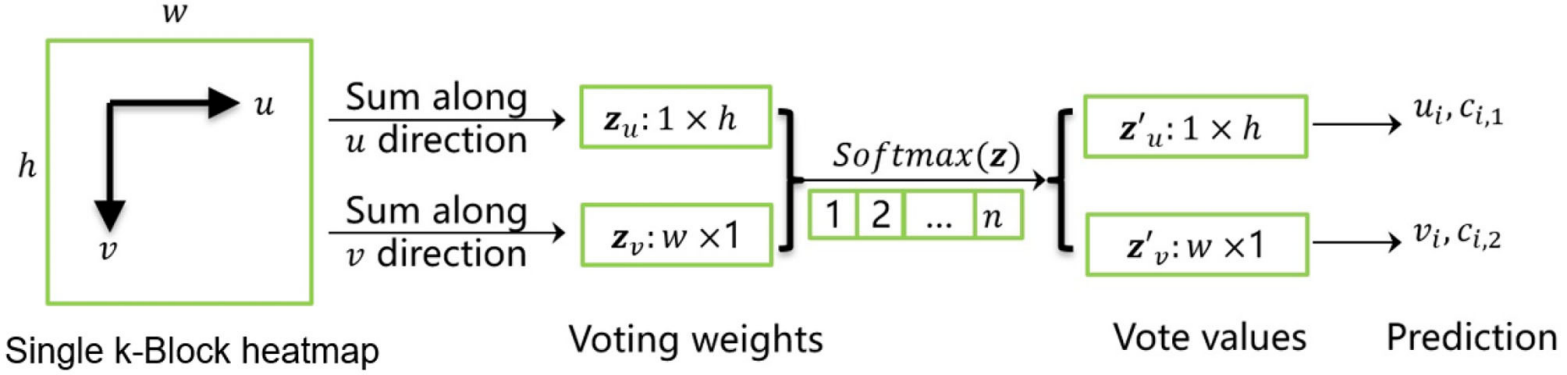
k-block prediction process.

To address these problems, we propose the k-block-based single-person pose estimation module, as illustrated in Figure 2. The input image is firstly processed by the backbone network to extract a feature tensor with a size of *w*×*h* pixels and *l* channels. With a further convolution in the channel dimension, a new tensor is predicted with *k* channels, which is equal to the number of to be predicted joint points. The tensor is further fed into the k-block module to generate the voting matrices. The joint points of the human body are finally predicted according to the corresponding voting results. The detailed calculation process is shown in Figure 3.

Here, we denote the *i*-th channel of input tensor as a heatmap (with a size of *w*×*h*). The k-block module firstly accumulates heatmap values in both *u*- and *v*-directions. The obtained vectors ***z***_*u*_ and ***z***_*v*_ are then considered as the coordinate voting weights of the corresponding joint point in the *u*- and *v*-direction. By applying the *Softmax* operation on both weighting vectors, the normalized weight distributions $z_{u}^{{}^{\prime }}$ and $z_{v}^{{}^{\prime }}$ are obtained. Given a vector with a length of *n*, it generates an enumeration vector ***e*** = [1, 2, ...*n*], which corresponds to the sequence of row or column IDs. The element-wise product of the normalized weight distribution z^*^ and the enumeration sequence ***e*** is thus the distribution of corresponding voting values. The predicted joint coordinates can be calculated by summing up of the voting values. Additionally, we denote the joint coordinate uncertainty *c*_*i,u*/*v*_ as the standard deviation of the voting values, i.e., the more concentrated the vote distribution is, the lower the uncertainty will be.

**Figure 4:**
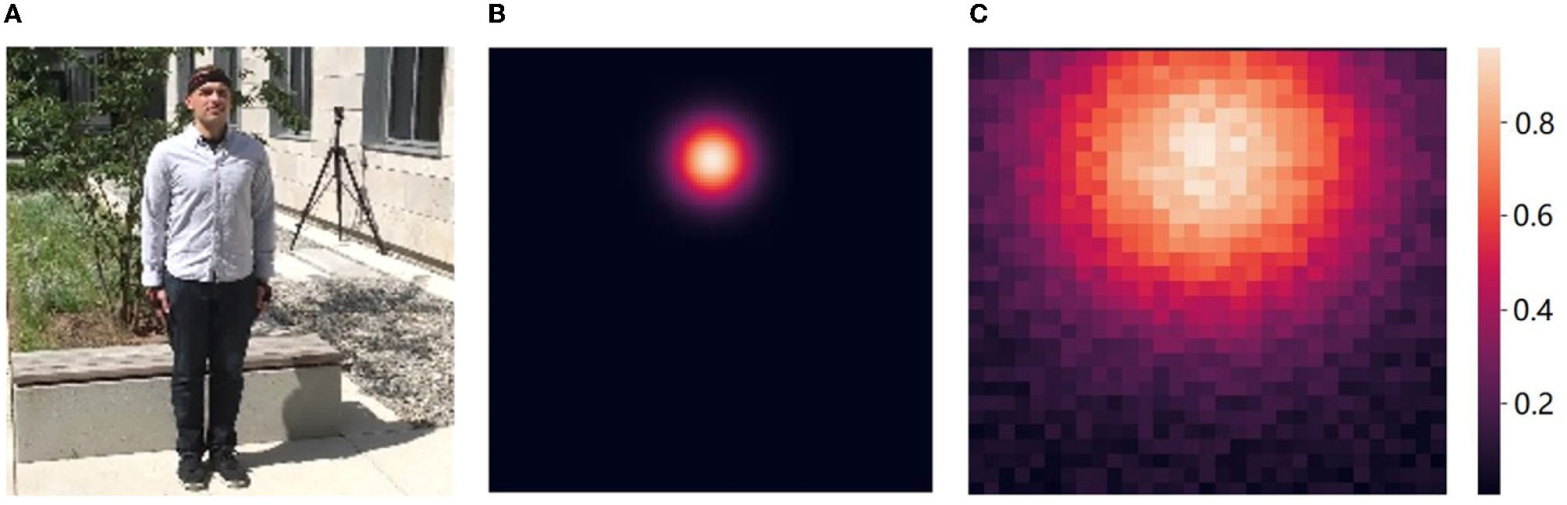
Comparison of Gaussian heatmap and k-block weights. **(A)** Input. **(B)** Gaussian (128 × 128). **(C)** k-Block (32 × 32).

A comparison of Gaussian heatmap used in traditional methods and the k-block weights predicted in our approach is illustrated in Figure 4. In order to achieve a sufficient accuracy for the joint location, Gaussian heatmaps often require a larger resolution (e.g., 128 × 128 pixels). The non-joint areas are indicated in black in Figure 4B, in which a large number of gradients are used to suppress non-zero predictions. This part of the gradients has little effect on the prediction of joint points, resulting in a slow convergence of the model. Moreover, it still consumes a lot of computation in these areas in the forward inference stage, although their predictions are not considered. However, for heatmaps with larger Gaussian kernels, although more pixels are involved in the joint point estimation, the location accuracy can be reduced due to the reduction of the gap between predicted values.

In this paper, a new k-block structure is designed and the coordinate values of human joints are calculated from all heatmap elements at the same time, which greatly reduces the waste of gradients based on Gaussian heatmap prediction, so that it can use less computation (e.g., with a resolution of 32 × 32 pixels, which is yet still larger than the small local joint region in Gaussian heatmap) to obtain more gradient propagation to achieve similar accuracy.

In our proposed approach, each joint point estimation is regarded as a Gaussian distribution. Given an estimated coordinate *x*_*i*_ (i.e., *u*_*i*_ or *v*_*i*_) and its ground truth ${\hat{x}}_{i}$, the estimation error *f*_*e*_(*x*_*i*_) follows the Gaussian distribution, interpreted as


(1) $$\begin{array}{l} f_{e}\left(x_{i}\right)=\frac{1}{\sqrt{2\pi }c_{i}}\exp\left(-\frac{{\left(x_{i}-{\hat{x}}_{i}\right)}^{2}}{2c_{i}^{2}}\right) \end{array}$$

with the standard deviation *c*_*i*_. By applying the logarithm form of (1) and considering all joint points, the loss for k-block module is expressed as


(2) $$\begin{array}{l} L_{KB}=\underset{i}{\sum }\left(\log\left(\sqrt{2\pi }c_{i}\right)+\frac{{\left(x_{i}-\hat{x}\right)}^{2}}{2c_{i}^{2}}\right)+\omega_{c}\underset{i}{\sum }\frac{1}{2}c_{i}^{2}, \end{array}$$

where ω_*c*_ represents the weight of the additional regularization term and is empirically set to 0.2. The set of inferred 2D joint points are denoted as P_2*D*_ = {***p***_2*D*, 1_, ..., ***p***_2*D, k*_}.

### 3.2. 3D human characterization based on SMPL parameters

The SMPL (Loper et al., 2015) is a vertex-based three-dimensional model containing a fixed set of parameterized expressions based on the statistics of a large amount of real human body data. In this paper, the SMPL model is selected as the prior structure of the rigid human body, since it can accurately express different postures and movements. It should be noted that the original SMPL model also needs a set of root coordinates to further determine the 3D coordinates of the joint point. In this paper, we focus on the spatial relation between the 3D coordinates (e.g., relative to the body center), thus it requires no additional corresponding root points. Here, we implement an additional output head after the decoder of Derformable DETR (Zhu et al., 2021) to infer both the human body shape parameter ***β*** and the pose parameter ***θ*** from an input image, as illustrated in the middle part of Figure 1.

The complete shape parameters consist of a total of 50 items with only the first 10 open-sourced. Statistics show that most of the parameter values are in the range from –1.5 to +1.5. This paper chooses the Smooth-L1 loss as the shape loss function and adjusts its second-order loss range to (–1.5, 1.5), interpreted as


(3) $$\begin{array}{l} L_{shape}=\sum_{i}\left\{ \begin{array}{l} \frac{2}{9}{\left(\beta_{i}-{\hat{\beta }}_{i}\right)}^{2},\;|\beta_{i}-{\hat{\beta }}_{i}|\;\leq 1.5\\ |\frac{2}{3}{\left(\beta_{i}-\hat{\beta }\right)}_{i}|-\;0.5,\;|\beta_{i}-{\hat{\beta }}_{i}|\;>1.5 \end{array}\right.\;, \end{array}$$

where β_*i*_ is the predicted *i*-th element of shape parameter ***β*** in the SMPL model and the symbol ˆ indicates the ground truth.

Additionally, we introduce the Quaternion notation to avoid the ambiguity problem induced by Euler angles used in the original SMPL. Let the normalized vector of the rotation axis be (*x*′, *y*′, *z*′) and the rotation angle be α∈(−π, π]. The pose parameter of SMPL can be expressed as


(4) $$\begin{array}{l} \theta =\left(x^{\prime }\sin\frac{\alpha }{2},y^{\prime }\sin\frac{\alpha }{2},z^{\prime }\sin\frac{\alpha }{2},\cos\frac{\alpha }{2}\right). \end{array}$$

Considering that the Quaternion representation is a normalized vector and its element value is in the range of (−1, 1), the loss function of the pose parameter is selected as an L1 loss with an additive regularization term:


(5) $$\begin{array}{l} L_{pose}=\|\theta -\hat{\theta }{\|}_{1}+\omega_{p}\left|1-\|\theta {\|}_{2}^{2}\right|, \end{array}$$

where ***θ***_*i*_ represents the *i*-th element of ***θ*** and ω_*p*_ denotes the weight of the regularization term and empirically set to 1.

Based on the inferred shape parameter ***β*** and pose parameter ***θ***, we can estimate the 3D joint point coordinates according to the SMPL model. The computation details can be referred to work (Loper et al., 2015). The point set is coordinate-normalized (by removing the mean and rescaling with the reciprocal of standard deviation) and denoted as 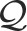
_3*D*_ = {***q***_3*D*, 1_, ..., ***q***_3*D, k*_}.

### 3.3. 2D-3D keypoint optimization

To correct the prediction results, especially for 2D joint points, we resort to the idea of 3D point matching. Generally, given two sets of matched 3D points P = {***p***_1_, ***p***_2_, ..., ***p***_*k*_} and 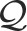 = {***q***_1_, ***q***_2_, ..., ***q***_*k*_}, the aim is to find a set of Euclidean transforms {***R, t***} to minimize their alignment errors. The optimal transform {R^*^, t^*^} can be obtained by solving the Least Squares problem as


(6) $$\begin{array}{l} (R^{*},t^{*})=\text{arg min}\sum_{i}^{k}\frac{1}{2}||Rp_{i}+t-q_{i}{|}_{|}^{22}. \end{array}$$

If the mean values of both sets P and 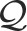 are removed, which means their center are aligned at the origin, we obtain


(7) $$\begin{array}{l} t^{*}=t=0. \end{array}$$

Thus, Eq. (6) can be reformulated as


(8) $$\begin{array}{l} R^{*}=\text{arg min}\sum_{i}^{k}\frac{1}{2}||Rp_{i}-q_{i}{|}_{|}^{22}. \end{array}$$

The square term of above equation can be calculated as


(9) $$\begin{array}{l} ||Rp_{i}-q_{i}|{|}_{2}^{2}=p_{i}^{⊤}p_{i}-p_{i}^{⊤}R^{⊤}q_{i}-q_{i}^{⊤}Rp_{i}+q_{i}^{⊤}q_{i}. \end{array}$$

Noting that ${\left(q_{i}^{⊤}Rp_{i}\right)}^{⊤}=p_{i}^{⊤}R^{⊤}q_{i}$, by discarding constant terms, Eq. (8) can be further simplified as


(10) $$\begin{array}{l} R^{*}=\text{arg max}\sum_{i}^{k}q_{i}^{⊤}Rp_{i}=\text{arg max tr}(Q^{⊤}RP)\;\\ \;=\text{arg max tr}(RPQ^{⊤}), \end{array}$$

where ***P*** and ***Q*** denote the matrix forms of point sets. Leveraging the SVD decomposition, it obtains ***PQ***^⊤^ = ***U*****Σ*****V***^⊤^. Equation (10) can then be reformed as


(11) $$\begin{array}{l} R^{*}=\arg\max\mathrm{tr}\left(RU\Sigma V^{⊤}\right)=\arg\max\mathrm{tr}\left(\Sigma V^{⊤}RU\right). \end{array}$$

Since ***R***, ***U***, and ***V*** are all orthogonal matrices, the matrix ***M*** = ***V***^⊤^***RU*** is also orthogonal. Thus, we obtain


(12) $$\begin{array}{l} 1=m_{i}^{⊤}m_{i}=\underset{j}{\sum }m_{i,j}^{2}\to m_{i,j}^{2}\leq 1\to |m_{i,j}|\leq 1, \end{array}$$

where ***m***_*i*_ is the *i*-th row of ***M*** and ***m***_*i,j*_ is the *j*-th element of ***m***_*i*_. As **Σ** = diag[σ_1_, ..., σ_*k*_] is a diagonal matrix, there is


(13) $$\begin{array}{l} \mathrm{tr}\left(\Sigma M\right)=\underset{i}{\sum }\sigma_{i}m_{i,i}\leq \underset{i}{\sum }\sigma_{i}. \end{array}$$

Obviously, only with ***m***_*i,i*_ = 1 can tr(**Σ*****M***) be maximized. Then, ***M*** becomes a unit matrix, which is


(14) $$\begin{array}{l} I=M=V^{⊤}R^{*}U. \end{array}$$

By solving the above equation, we obtain the optimal rotation matrix R^*^ = ***VU***^⊤^.

If the depths of 2D joint points are known, with the above solution, we can correct the 2D joint points with their corresponding 3D coordinates estimated by the SMPL model, as illustrated in the right part of Figure 1. This is based on the fact that the SMPL is built on the statistics of a large set of real human bodies. Thus, its representation about the spatial relation between joint points should be more consistent with the real ones compared to the k-block-based estimation. Since the predicted 2D joint points are depthless, we consider their depths as additional to be optimized parameters in the entire optimization process. The main idea is to firstly lift the 2D key points into 3D space by assigning them with initial depth values, which are then gradually optimized by the 3D matching according to the solved rotation matrix. With iterations in this process, the accuracy of the estimated depth, the solved rotation matrix and the corresponding 2D coordinates of 3D key points are progressively improved. Here the *z*-axis is defined as aligned with the depth direction, which is perpendicular to the image plane.

During the optimization, we also introduce the uncertainties of estimated 2D keypoint locations by the k-block module. Since the joint points in occluded or low-light areas are often estimated more inaccurately due to less information, their uncertainties will be high and their matching errors should be less weighted. Thus, Eq. (8) can rewritten as


(15) $$\begin{array}{l} R^{*}=\arg\min\underset{i}{\sum }\frac{1}{2}w_{i}||Rp_{i}+q_{i}|{|}^{2}. \end{array}$$

The weight *w*_*i*_ is set to 1/*c*_*i*_, which is the reciprocal of the uncertainty *c*_*i*_. We further define a diagonal weight matrix ***W*** = diag[*w*_1_, ..., *w*_*k*_]. Leveraging Eq. (10), the above equation can be reformed as


(16) $$\begin{array}{l} R^{*}=\arg\max\mathrm{tr}\left(RP^{⊤}WQ\right)=\arg\max\mathrm{tr}\left(\text{Σ}V^{⊤}RU\right) \end{array}$$

with the new SVD decomposition ***P***^⊤^***WQ*** = ***U***Σ*V*^⊤^. This can be considered as a weighted 2D coordinate correction process based on SMPL parameters. Detailed steps of this process are listed in Algorithm 1, where the iteration number is empirically set to 3.

**Algorithm 1:**
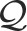
Weighted 2D coordinate correction based on SMPL parameters.

### 3.4. Multi-person detection and pose estimation

**Figure 5:**
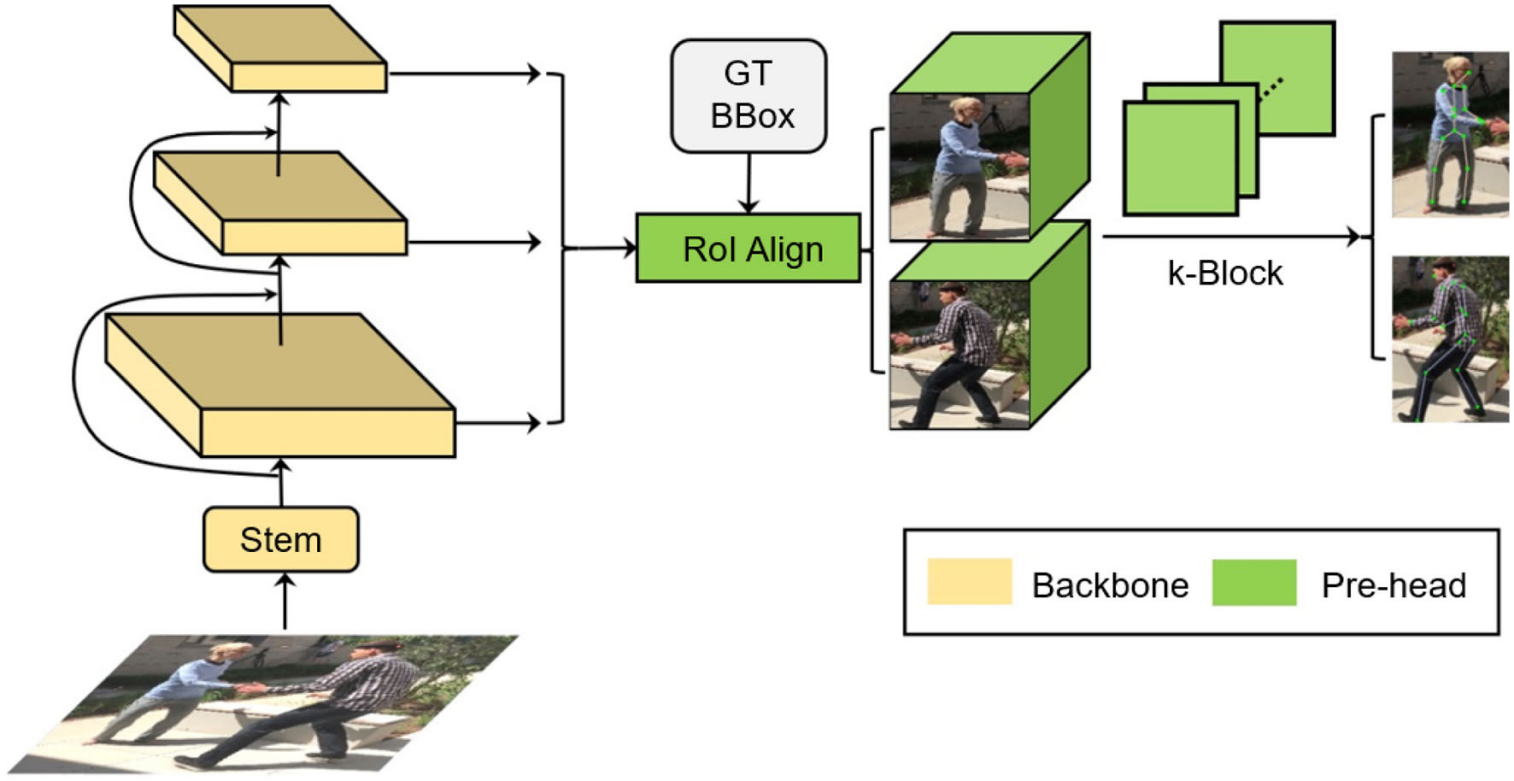
Multi-scale information for multi-person pose estimation.

Since pedestrians can appear in the image with different scales due to their sizes or distances in the 3D world space, the representation ability of features only extracted from a single layer of neural network becomes insufficient. Hence, the multi-person pose estimation scheme should be adapted to multi-scale image information. Considering the multi-layer convolution characteristics of the deep neural network itself, the deeper the layer is, the greater information amount a single neuron will capture, i.e., a deeper layer corresponds to a greater receptive field. Therefore, we can extract features from different layers of the backbone network to obtain the multi-scale information, as shown in Figure 5. Although such a multi-scale feature manipulation yields mere computational overhead, it has shortcomings like that the features from shallow layers are with relative low semantic information, limiting the prediction performance, while the deep layers are with relatively low resolution, leading to insufficient information amount within an RoI.

**Figure 6:**
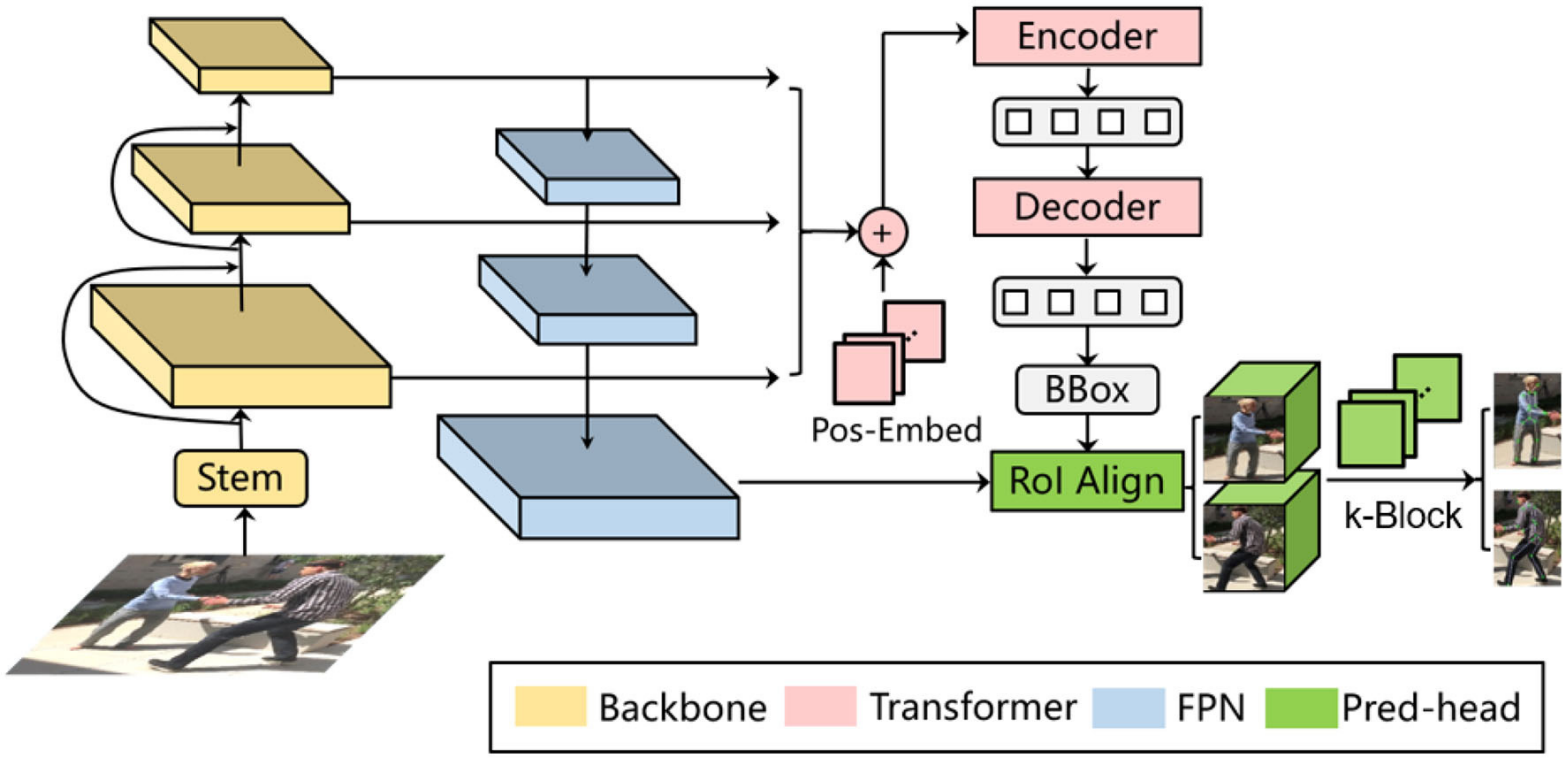
Deformable DETR-based multi-person pose estimation.

Referring to the Feature Pyramid Network model (FPN), we add an additional information recovery branch to the backbone (i.e., the ResNet). As shown on the left side of Figure 6, the bottom-up process indicates the feedforward feature calculation in the original model. As the layer deepens, the corresponding feature map gradually becomes downsampled. The top-down process is the gradual feature restoration toward the original image size. By fusing the information from different levels, the shallower layer obtains both higher resolution and richer semantic features. For inference, according to bounding box sizes, feature maps from the corresponding FPN layer are selected to be cropped and sent to the k-block module to estimate the pose of each individual person. Additionally, we adopt the RoI Align (He et al., 2017) to avoid the dislocation of feature tensors caused by quantization operations.

To further improve the pedestrian detector performance, we employ the Deformable DETR framework (Zhu et al., 2021), as illustrated in Figure 6. In terms of single-frame pose estimation, the Deformable DETR model provides the candidate regions of detected persons and their corresponding image features for the k-block module. Thus, a simultaneous multi-person detection and pose estimation can be achieved. In addition to the detection bounding boxes, we also introduce another output head to the original Deformable DETR to regress the shape and pose parameters ***β*** and ***θ*** of the SMPL model. The SMPL model is further applied in the iterative optimization process introduced in Section 3.3 to correct the predicted 2D key points, resulting in the final architecture proposed in this paper as shown in Figure 1. The total loss function for training the entire architecture is interpreted as


(17) $$\begin{array}{l} L_{total}=\;{\text{λ}}_{shape}L_{shape}+{\text{λ}}_{pose}L_{pose}+{\text{λ}}_{SMPL}L_{SMPL}+\;\\ {\text{ λ}}_{KB}L_{KB}+{\text{λ}}_{DET}L_{DET}, \end{array}$$

where *L*_*DET*_ denotes the object detection loss defined in the Deformable DETR (Zhu et al., 2021), *L*_*SMPL*_ represents the squared errors of keypoint coordinates predicted by the SMPL, and the subscripted term λ indicates the corresponding weight of each loss.

## 4. Experiments and evaluations

### 4.1. Experimental setups

Here we choose two mainstream datasets, i.e., 3DPW (Von Marcard et al., 2018) and Human3.6M (Ionescu et al., 2011, 2014), for experiments. The 3DPW is a single-view multi-person 3D pose dataset containing 60 video sequences (24 for training, 24 for test, and 12 for validation) shot in outdoor environments such as forests, streets, playgrounds, etc. This dataset also includes a large number of 2D/3D pose annotations, 3D body scans, and SMPL parameters. The Human 3.6M is a multi-view single-person 3D pose dataset captured in an indoor space. It contains 3.6 million 3D human poses and corresponding videos (50 FPS) from 15 scenes, with keypoint annotations of both 2D/3D positions and angles. For evaluation, the video is downsampled at a ratio of 5/64 to eliminate redundancy.

**Figure 7:**
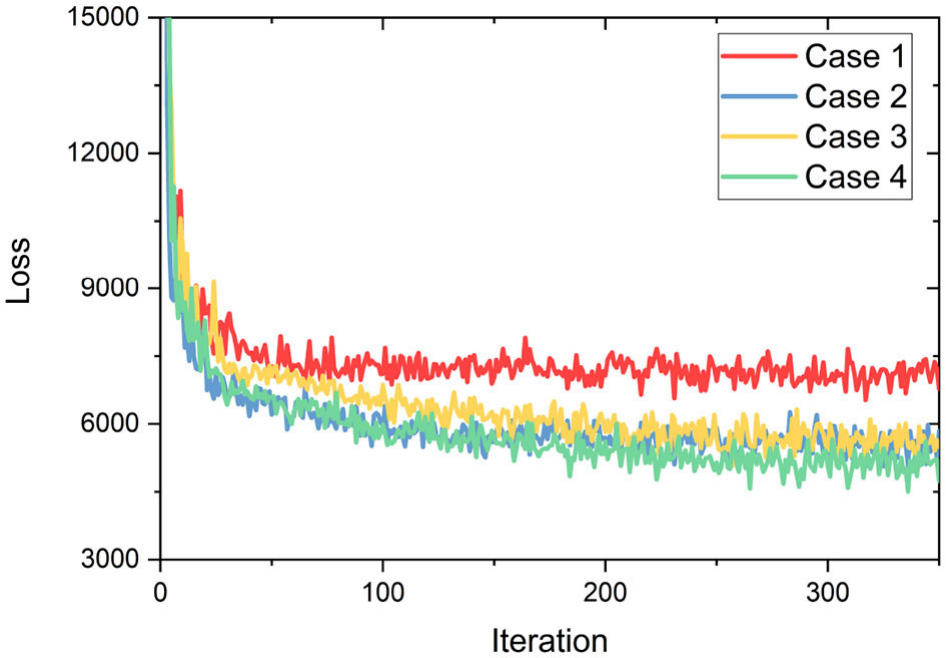
Trend of loss with different hyperparameters set (Case1: λ_*shape*_ = 0.4, λ_*pose*_ = 0.4, λ_*SMPL*_ = 0.4, λ_*DET*_ = 0.25, λ_*KB*_ = 0.25; Case2: λ_*shape*_ = 0.25, λ_*pose*_ = 0.25, λ_*SMPL*_ = 0.25, λ_*DET*_ = 0.25, λ_*KB*_ = 0.25; Case3: λ_*shape*_ = 0.25, λ_*pose*_ = 0.25, λ_*SMPL*_ = 0.25, λ_*DET*_ = 0.25, λ_*KB*_ = 0.4; Case4: λ_*shape*_ = 0.2, λ_*pose*_ = 0.25, λ_*SMPL*_ = 0.15, λ_*DET*_ = 0.4, λ_*KB*_ = 0.3).

Since our proposed method adopts predicted 3D key points to assist the correction of predicted 2D keypoint coordinates, 3D annotations are employed in supervising the module for 3D keypoint prediction learning, which is also one of the main reasons in choosing above datasets for evaluation. In experiments, the proposed architecture is implemented by the PyTorch on a computer platform with a CPU of Intel i9@3.50 GHz, a GPU of NVIDIA RTX 3090 and a memory of 32 GB. During training, we adopt the Adam optimizer with a learning rate of 1e-3. The manual selection of hyperparameters, based on experience, has a substantial effect on the outcome of training. Consequently, various hyperparameters were designed and promptly evaluated with a consistent number of iterations in order to choose the suitable configuration. It can be seen in Figure 7 that when the weights λ_*shape*_, λ_*pose*_, and λ_*SMPL*_ of 3D pose estimation are relatively small and the weight λ_*DET*_ of the human detection box is relatively large, there is a minimum loss trend (case 4). This may be due to the fact that the human detection box is the foundation of the top-down approach and its accuracy will directly influence the subsequent 2D/3D pose estimation. To this end, the weights for loss terms are empirically set as: λ_*shape*_ = 0.2, λ_*pose*_ = 0.25, λ_*SMPL*_ = 0.15, λ_*DET*_ = 0.4 and λ_*KB*_ = 0.3.

For evaluation, we choose metrics of Mean Per Joint Position Error (MPJPE) and the Procrustes Alignment Mean Per Joint Position Error (PA-MPJPE), calculated as follows:


(18) $$\begin{array}{l} MPJPE=\frac{1}{k}\overset{k}{\underset{i}{\sum }}||p_{i}-{\bar{p}}_{i}|{|}_{2}, \end{array}$$

(19) $$\begin{array}{l} PA-MPJPE=\frac{1}{k}\overset{k}{\underset{i}{\sum }}||p_{i}^{\prime }-{\bar{p}}_{i}^{\prime }|{|}_{2}, \end{array}$$

where ***p***_*i*_ refers to the predicted position of the *i*-th joint point while ${\bar{p}}_{i}$ indicates the corresponding ground truth. The $p_{i}^{\prime }$ also denotes the position of the *i*-th joint point, yet with the predicted skeleton firstly aligned to its ground truth by rotation, translation and scaling. To facilitate a fair comparison with other mainstream pose estimators on above benchmarks, we calculate the corresponding 3D coordinates of predicted 2D key points by using the optimized depths and the given camera parameters. Thus, position errors can be measured in the 3D space.

### 4.2. Evaluation on multi-person pose estimation

In the first experiment, we explore the performance of different strategies for multi-person pose estimation introduced in Section 3.4, i.e., the direct multi-scale information fusing scheme, the FPN-based scheme and the SMPL correction-based scheme. For a fair comparison, all schemes adopt the Deformable DETR as base-detector and are evaluated on the 3DPW dataset. The results are reported w.r.t. the MPJPE metric in Table 1.

**Table 1:** Exploration on performance of different multi-person pose estimation strategies with ↓ indicating that lower values are better.

**Multi-scale**	**FPN**	**Correction**	**MPJPE (mm)↓**
✓			58.5
	✓		57.9
	✓	✓	57.2

**Figure 8:**
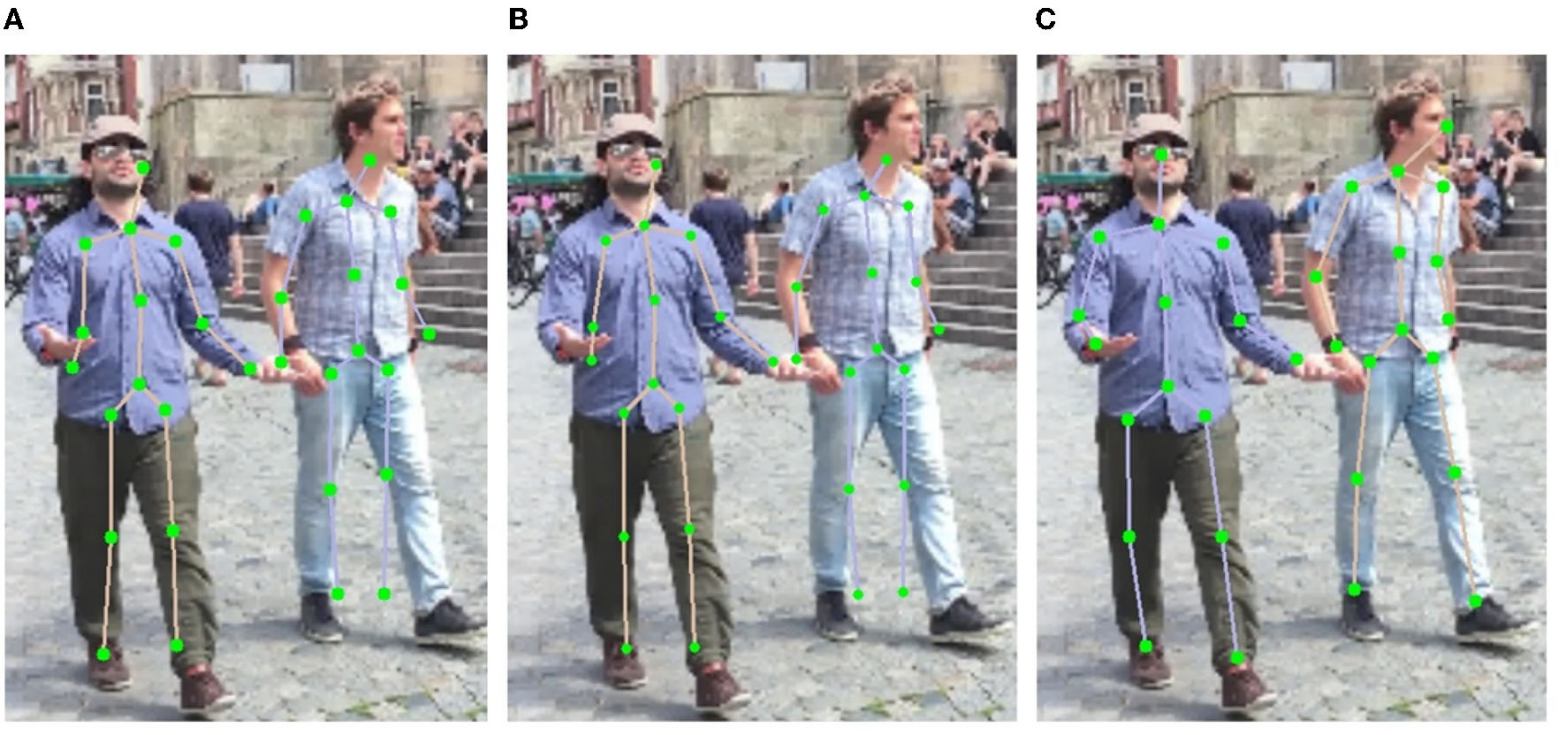
Examples of results under different multi-person pose estimation strategies. **(A)** Multi-scale. **(B)** FPN. **(C)** FPN and correction.

Obviously, introducing FPN module improves the mean joint position error by 0.6 mm according to the MPJPE metric, which proves that the top-down feature restoration process in the FPN is more efficient than the direct feature combination of different scales. By integrating the SMPL correction algorithm, the MPJPE is further reduced by 0.7 mm, demonstrating the benefit of 3D human body structure prior in the 2D keypoint prediction task. The processing speed of our entire architecture is about eight–nine FPS, which can be applied in real-time use cases. A qualitative comparison is also shown in Figure 8. As depicted, the direct multi-scale information fusion yields relative large estimation errors (Figure 8A). By only introducing the FPN module, the improvement is limited (Figure 8B). By further deploying the SMPL correction algorithm, the estimation errors at the end of the torso, on the arms and on the legs are compensated (Figure 8C).

**Figure 9:**
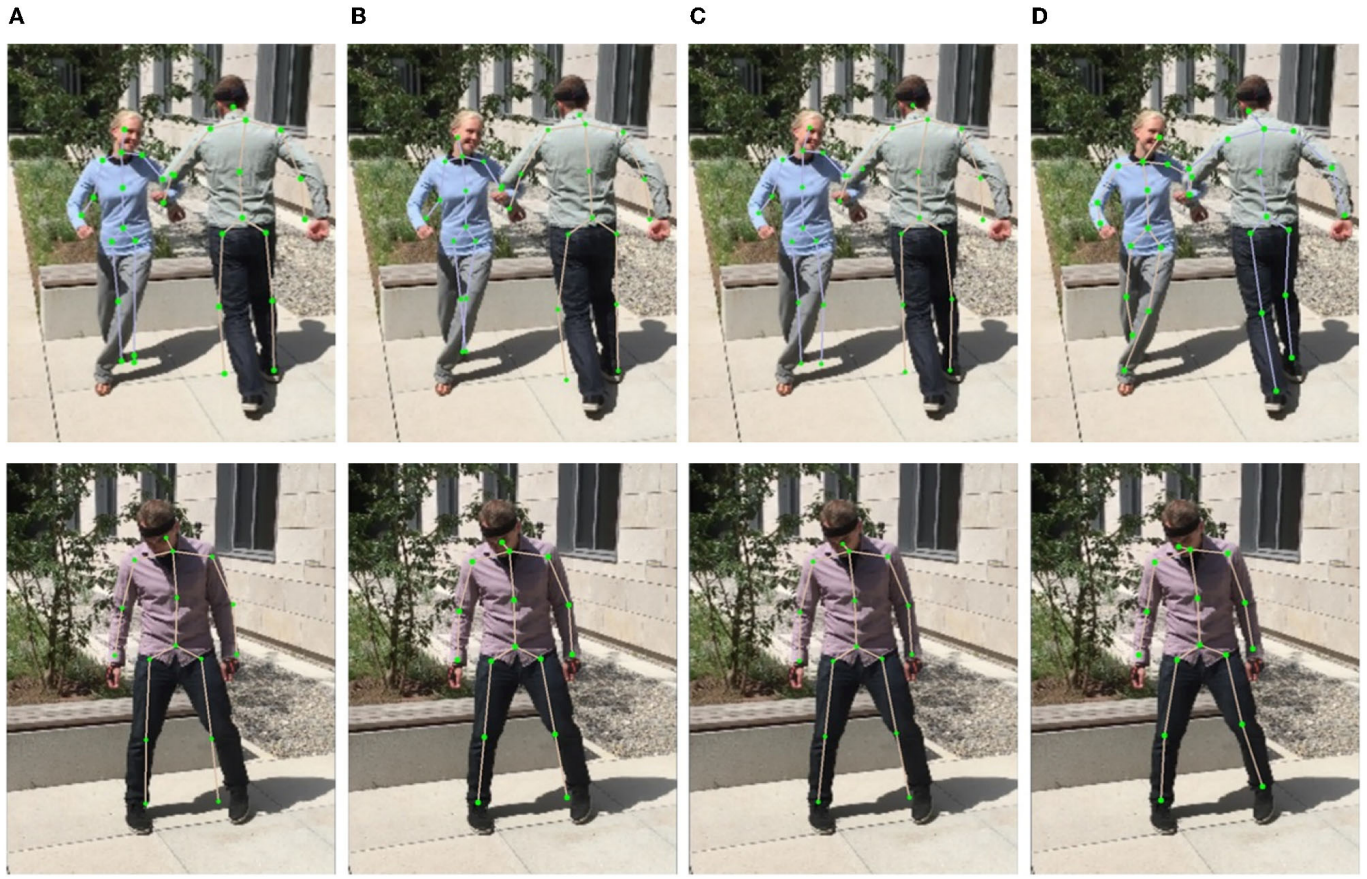
Example of prediction results by different multi-person pose estimators. **(A)** HMR. **(B**) SPIN. **(C)** HybrIK. **(D)** Ours.

We also compare the pose estimation results of our proposed architecture with those by other mainstream multi-person pose estimators including HMR (Kanazawa et al., 2018), SPIN (Kolotouros et al., 2019), ROMP (Sun Y. et al., 2021), HybrIK (Li et al., 2021) and DynBOA (Huang et al., 2020). Results of compared methods are listed in Table 2. It can be seen that the model based on k-block and SMPL parameter estimation proposed in this paper has reached a new level of state-of-the-art performance on the 3DPW dataset. It outperforms other approaches by an error reduction of about 5 mm w.r.t. the PA-MPJPE metric. In terms of the MPJPE metric, a larger accuracy gain is obtained, which is 8.3 mm. Examples of pose prediction results are shown in Figure 9. To be noted, since some of compared methods are not open-sourced, we only illustrate the prediction results of methods whose codes are available. As can be seen, in complex activities such as couple dancing, the key points at the end of body parts (e.g., arms and legs) can be easily misdetected in mainstream pose estimators while our method can still accurately locate these key points, proving its strong scene adaptability. Furthermore, we compare the inference time of the proposed method to the published results of other approaches, whose specific results are presented in Table 3. The use of DETR, with its large number of network parameters, inevitably sacrifices inference speed in order to achieve good results.

**Table 2:** Comparison with state-of-the-art multi-person pose estimators.

**Model**	**MPJPE (mm)↓**	**PA-MPJPE (mm)↓**
HMR (Kanazawa et al., 2018)	130.0	81.3
SPIN (Kolotouros et al., 2019)	96.9	59.2
ROMP (Sun Y. et al., 2021)	76.7	47.3
HybrIK (Li et al., 2021)	74.1	45.0
DynBOA (Huang et al., 2020)	65.5	40.4
Ours	57.2	35.5

**Table 3:** Runtime comparisons with different estimators.

**Method**	**FPS**	**Backbone**	**Device**
RepNet (Wandt and Rosenhahn, 2019)	10	Stacked hourglass network	NVIDIA TITAN X
VIBE (Kocabas et al., 2020)	10.9	ResNet-50	1070Ti GPU
ROMP (Sun Y. et al., 2021)	20.8	HRNet-32	1070Ti GPU
ROMP (Sun Y. et al., 2021)	30.9	ResNet-50	1070Ti GPU
Ours	9	DETR	NVIDIA RTX 3090

### 4.3. Evaluation on single-person pose estimation

Although our proposed architecture is designed aiming at the multi-person pose estimation task, it can still be applied for single-person pose estimation. Here, we evaluate our architecture on the Human3.6M dataset. As this dataset consists of millions of images and our computation resources are limited, we train our approach only on 10% of the training set. The evaluation results are reported in Table 4. As can be seen, the video-based pose estimators generally outperform single-view-based approaches. This can be attributed to additional motion information extracted from consecutive frames. However, the increased accuracy comes at the cost of processing a large number of frames, such as the top-ranked method Att3DPose, which requires 243 input frames. As to our method, its performance is comparable to the video-based VIBE (Kocabas et al., 2020) and Bundle (Arnab et al., 2019), and surpasses the singe-view-based RepNet (Wandt and Rosenhahn, 2019) and SMPLify (Bogo et al., 2016). Although the SMPLify is also an SMPL-based model, we achieve a position error reduction of about 15 mm by adopting the iterative optimization of 2D–3D key points, further demonstrating its advantages. However, our method is still with an error gap of 9 mm to the method HMR (Kanazawa et al., 2018), which is learned on half of the training data. As our model is only learned on 10% of the training data, there is still potential to improve its performance.

**Table 4:** Comparison with state-of-the-art single-person pose estimators.

	**Model**	**MPJPE (mm)↓**	**Input frames**	**Training ratio**
Video	VIBE (Kocabas et al., 2020)	65.6	16	50%
	Bundle (Arnab et al., 2019)	63.3	190	100%
	Att3DPose (Liu et al., 2020)	45.1	243	100%
Img.
Single
Img.
RepNet (Wandt and Rosenhahn, 2019)	89.9	1	100%
	SMPLify (Bogo et al., 2016)	80.7	1	50%
	HMR (Kanazawa et al., 2018)	56.8	1	50%
	Ours	65.8	1	10%

### 4.4. Exploration on uncertainty weighting

**Figure 10:**
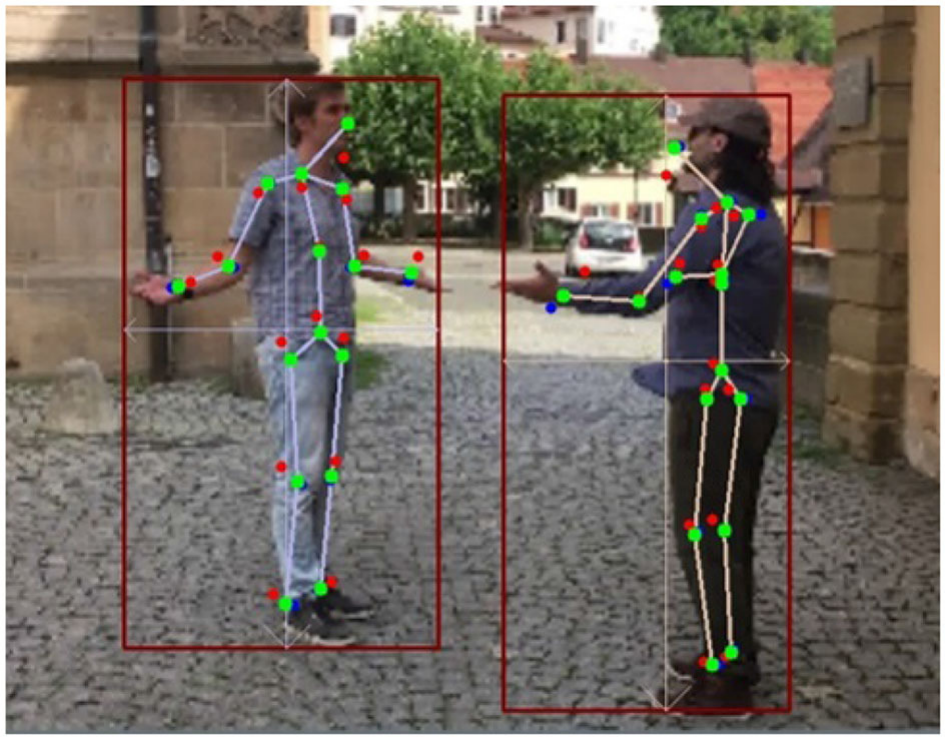
Example of multi-person pose estimation results (Red points: original output coordinates of the k-block module; Blue points: results by unweighted 3D correction algorithm; Green points: results by uncertainty-based weighted correction algorithm).

**Figure 11:**
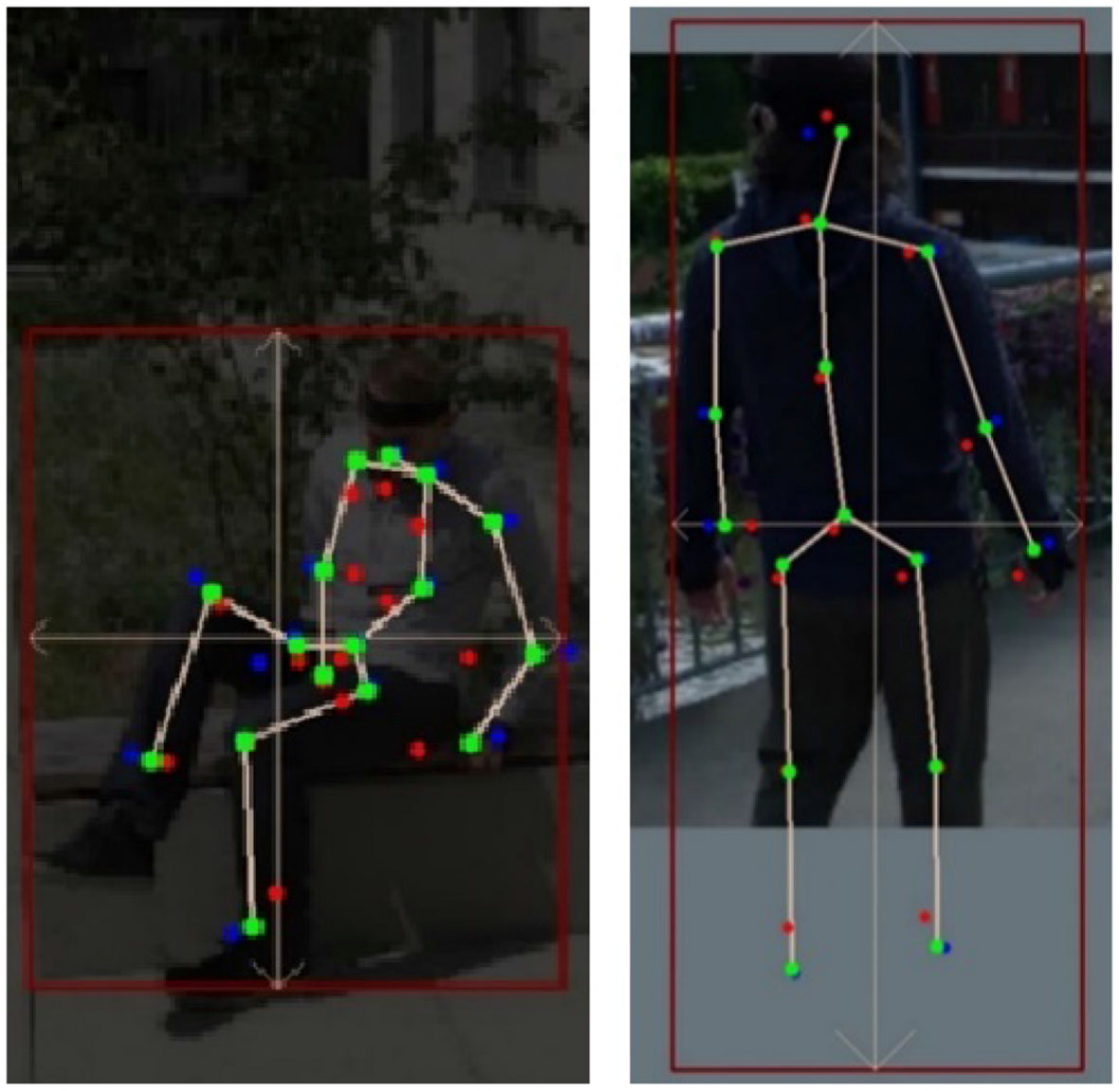
Prediction results under low-illumination **(left)** and with occlusion **(right**).

The essence of k-block module is not only to predict the 2D key points but also to estimate their uncertainties based on the large heatmap information. In this experiment, we qualitatively explore its influence on the keypoint weighting in the optimization process. As illustrated in Figure 10, we depict the key points directly predicted by the k-block module in red, the ones corrected by SMPL yet without considering uncertainties in blue, and those corrected by the uncertainty-based weighted optimization in green. As can be seen, key points directly predicted by the k-block module are with obvious errors such at the head, elbows, writs, and ankles. By applying the correction algorithm with the 3D SMPL model, the keypoint errors at the end of body parts are only reduced to some extent (e.g., the hand of the right person in Figure 10). By introducing uncertainty-based weighting in the optimization process, the keypoint errors are further reduced and the estimated skeleton looks more realistic. The uncertainty-based weighting is also beneficial to use cases under low-illumination or with occlusion, where individual key points become difficult to predict due to deteriorated image information. However, by considering uncertainties in the optimization, we can still obtain relative accurate keypoint prediction by fitting the informative body parts with the 3D shape and pose estimated by the SMPL model (Figure 11), validating the proposed approach.

## 5. Conclusion and discussion

**Figure 12:**
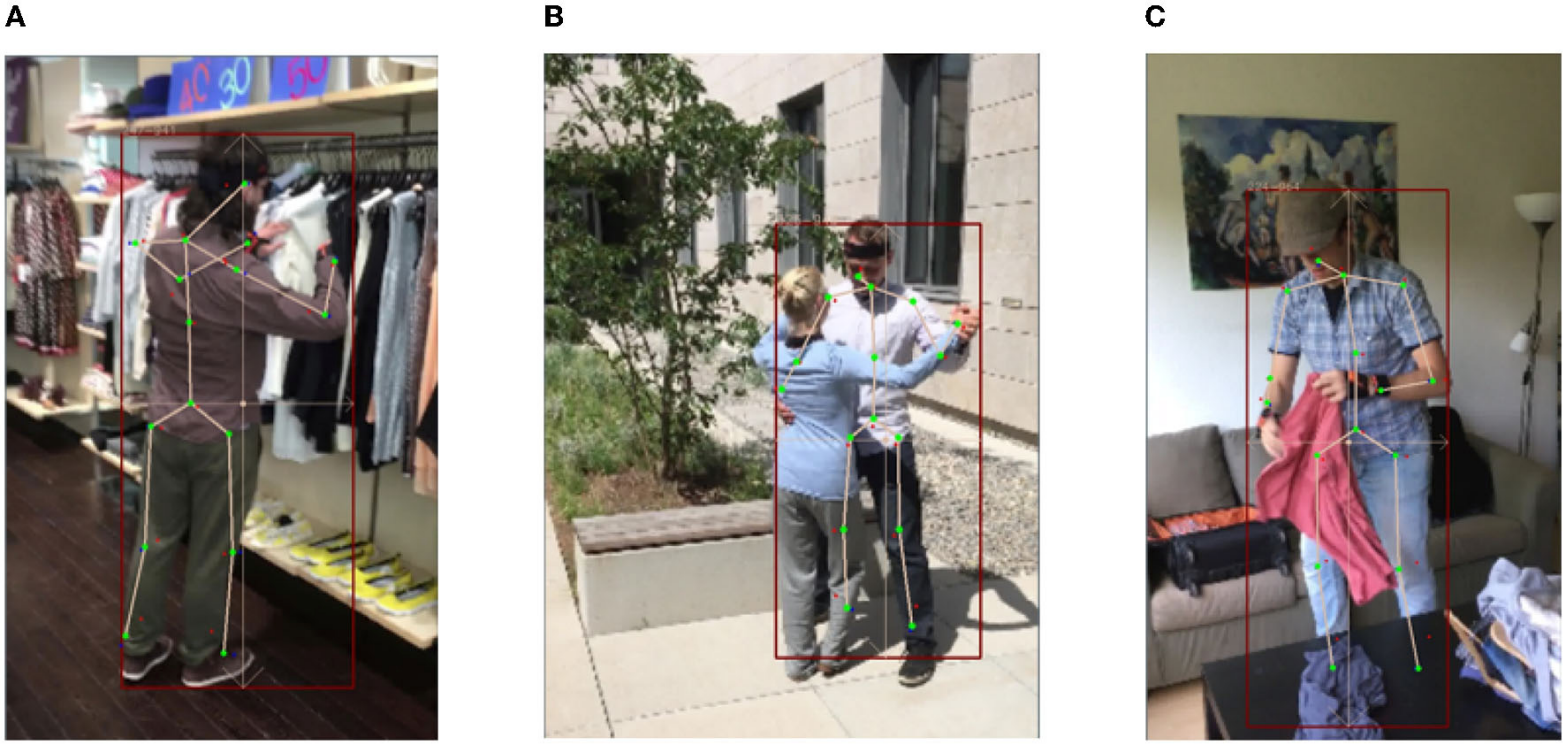
Example of negative results caused by occlusion with significant errors on legs. **(A)** Self-occlusion. **(B)** Occlusion by other people. **(C)** Occlusion by object.

In this paper, we present a new single-view multi-person pose estimation approach. It manifests improvements over existing approaches in two main aspects: Firstly, it proposes a k-block module to simultaneously calculate the 2D key point coordinates and their uncertainties, which improves the extraction of heatmap features and facilitates the attentive learning of more informative key points. Secondly, it employs a 3D shape and pose estimation based on the SMPL model and further proposes an uncertainty-weighted correction algorithm to iteratively align the estimated 3D coordinates with the predicted 2D key points. By experiments on the 3DPW benchmark, it surpassing state-of-the-art approaches by a gain of about 8 mm on MPJPE metric and 5 mm on PA-MPJPE metric. Additionally, it is real-time applicable and preforms robust against complex scenarios. Nonetheless, when the human body is subjected to self-occlusion or occlusion (see Figure 12), there is an ambiguity in depth estimation, which has a consequential impact on 3D pose estimation. Therefore, it is worth noting several important considerations for the future work: (1) incorporating an angle-axis representation or a regularization term to represent rotation; (2) improving the model accuracy for node coordinates through the utilization of multi-perspective images and designing a lighter, more compact model through network coding schemes.

## Data availability statement

The original contributions presented in the study are included in the article, further inquiries can be directed to the corresponding author.

## Author contributions

WT contributed to the conceptualization, methodology, supervision, writing, and review of the paper. ZG contributed to the methodology, experiments, and writing of the paper. DT contributed to the methodology, writing, and review of the paper. All authors contributed to the article and approved the submitted version.
